# Establishment of a multi-parameter prediction model for the functional cure of HBeAg-negative chronic hepatitis B patients treated with pegylated interferonα and decision process based on response-guided therapy strategy

**DOI:** 10.1186/s12879-023-08443-1

**Published:** 2023-07-10

**Authors:** Qianqian Tang, Jun Ye, Yafei Zhang, Peixin Zhang, Guomei Xia, Jie Zhu, Shaofeng Wei, Xu Li, Zhenhua Zhang

**Affiliations:** 1 Department of Infectious Diseases, The Second Affiliated Hospital of Anhui Medical University, Furong Road 678, Hefei, 230601 Anhui China; 2 Department of Infectious Diseases, The First Affiliated Hospital of Anhui Medical University, Hefei, China

**Keywords:** Chronic hepatitis B, HBeAg-negative, Hepatitis B surface antigen, Score model, PEG-IFNα, RGT

## Abstract

**Background & aims:**

This study aimed to establish multivariate prediction models according to a response-guided therapy (RGT) based strategy at baseline and week 12 and 24 of follow-up to predict the functional cure for HBeAg-negative patients with chronic hepatitis B (CHB) treated with pegylated interferonα (PEG-IFNα).

**Methods:**

A total of 242 HBeAg-negative patients with CHB were treated with PEG-IFNα for 52 weeks and followed up for 24 weeks. Responses at the end of follow-up (EOF) were defined as hepatitis B surface antigen (HBsAg) loss, and patients were defined as either responders or non-responders.

**Results:**

The three most meaningful predictors were an age ≤ 40 years, alanine aminotransferase (ALT) levels ≤ 40 U/L, and HBsAg levels ≤ 100 IU/mL at baseline; ALT levels ≥ 80 U/L, anti-HBc levels ≤ 8.42 S/CO, and HBsAg levels ≤ 50 IU/mL at week 12; and ALT levels ≥ 40 U/L, anti-HBc levels ≤ 8.46 S/CO, and HBsAg levels ≤ 0.2 IU/mL at week 24. The response rates of patients with a score of 0–1 and 4–5 at baseline, week 12, and 24 were 13.5%, 7.8%, and 11.7%; and 63.6%, 68.1%, and 98.1%, respectively. At week 12, the cumulative scores were 0–2, 3–4, 5–7, and 8–10 (response rates 5.0%, 18.9%, 41.3%, and 71.4%, respectively). At week 24, the cumulative scores were 0–3, 4–6, 7–10, and 11–15 (response rates: 1.3%, 12.3%, 37.0%, and 92.5%, respectively). At baseline, patients with scores of 0–1 were slightly recommended; at week 12, patients with 0–1 or 0–2 cumulative scores were recommended to stop treatment. At week 24, patients with a score of 0–1 or a cumulative score of 0–6 were recommended to stop treatment.

**Conclusion:**

We established a multi-parameter prediction model for the functional cure of HBeAg-negative patients with CHB treated with PEG-IFNα.

**Supplementary Information:**

The online version contains supplementary material available at 10.1186/s12879-023-08443-1.

## Introduction

Chronic hepatitis B virus infection is a significant worldwide health problem affecting approximately 257 million people [1–5]. It may develop into cirrhosis, hepatic decompensation, hepatocellular carcinoma, and hepatic failure [6, 7]. HBsAg and HBV DNA levels have been reported to be closely associated with disease progression [8–10]. Interferon and nucleoside analogs (NUCs) are currently used as antiviral agents for treating CHB [11–13]. Antiviral treatment plays an important role in alleviating disease progression, with the desired endpoint of treatment being HBsAg seroclearance, referred to as “functional cure” [14–16].

NUCs have been demonstrated to be well-tolerated and generally safe [10]. They are effective for suppressing HBV replication, but not for silencing or eliminating circular DNA; hence, they hardly reduce HBsAg levels, and the overall therapeutic effect is unsatisfactory. Moreover, long-term NUCs antiviral therapy has potential drug-related side effects, psychological burden, relapse from drug withdrawal, and risk of virus resistance [17–19]. IFN exerts direct antiviral and immunoregulatory activities, inhibiting HBV DNA and RNA replication and eliminating virus-infected cells by enhancing the activity of immune cells, including that of cytotoxic T cells and antigen presenting cells, which in turn inhibit viral protein synthesis and the maturation and egress of virus particles. Moreover, PEG-IFNα has the advantages of a shorter therapy course, lower drug resistance rate, higher HBsAg clearance/seroconversion rates, and a once-weekly administration rate compared with conventional drugs, with the last providing improved adherence [20–23].

However, there is a limitation to the clinical utility of PEG-IFNα due to its side effects, and only a small percentage of patients reach functional cure. Moreover, PEG-IFNα therapy is expensive and has several contraindications [24, 25]. Therefore, it is a significant challenge to predict whether patients will benefit from PEG-IFNα therapy at the early stages of treatment course. Several recent studies have shown that ALT, HBsAg, anti-HBc, HBV DNA, HBV genotype, and other parameters can be used to predict HBsAg seroclearance in patients with CHB treated with PEG-IFNα [26–32]. Dynamic changes in HBsAg levels have been identified as being predictive of IFN treatment response in past researches [33, 34]. Nonetheless, these studies have ordinarily concentrated on single parameters or baseline prediction or were conducted in a single center. Moreover, there were significant differences in PEG-IFNα treatment regimens among the different studies, as well as significant differences in predictive model parameters. The predictive power was also very limited.

We followed 267 HBeAg-negative patients with CHB treated with PEG-IFNα in two centers. A logistic regression analysis assessed the performance of multiple parameters at baseline and throughout the therapeutic course at predicting HBsAg loss at EOF; the cutoff values of clinically useful parameters were used to construct prediction models. We developed prediction models for PEG-IFNα response in HBeAg-negative patients with CHB, who achieved functional cure, and developed a clinically practical treatment decision-making process following a RGT-based strategy.

## Methods

### Patients

This study was a retrospective study carried out in two medical centers. A total of 267 HBeAg-negative patients with CHB receiving PEG IFNα treatment in the first and second affiliated hospitals of Anhui Medical University from May 2015 to June 2022 were enrolled in the study. The inclusion criteria were at least 6 months of HBsAg positivity, negative HBeAg and anti-HBs, and initial or experienced treatment with PEG- IFNα monotherapy or combination therapy. The exclusion criteria were co-infection with hepatitis C virus, human immunodeficiency virus, or other viruses; decompensated liver disease or neoplasms of the liver; neutrophil count < 1.0 × 10^9^/L or platelet count < 5.0 × 10^9^/L; alcoholism and pregnancy. The study was approved by the Ethics Committee of Anhui Medical University (approval number: 2,012,624), and written informed consent was obtained from all patients.

One 180 µg dose of PEG-IFNα (PEG-IFNα-2a, Roche Pharmaceuticals, Shanghai, China or PEG-IFNα-2b, Amoytop Biotech, Xiamen, China) was injected weekly subcutaneously, with a subsequent 24-week period of follow-up. A sample of patients treated for at least 12 weeks was analyzed. For the few patients lost during follow-up or who changed therapy during PEG IFN treatment, laboratory parameters from the last visit were analyzed. The primary endpoint was loss of HBsAg 24 weeks after EOF.

### Laboratory measurements

Serum samples were tested at baseline (the most recent data available (within a month) for patients before treatment with PEG-IFN), over the treatment period (week 12, 24, and 52), and at the EOF in a central laboratory. An automatic biochemical analyzer (Roche, Basel, Switzerland) was used to measure serum ALT levels, and results are presented as multiples of the upper limit of normal (40 U/L). TaqMan-based real-time polymerase chain reaction assays (Shanghai FX MedTech, Shanghai, China) were used to measure HBV-DNA levels, with a quantification limit of 500 copies/mL. Commercially available enzymatic immune assays (Abbott, Chicago, IL, USA) were used to determine HBV serological markers (HBsAg, anti-HBs, HBeAg, anti-HBe, and anti-HBc).

### Recommendations and standards

This survey was developed based on the opinions of expert and medical personnel through a small-scale survey. Recommendation was given according to prospective HBsAg seroclearance rates at EOF for HBeAg-negative patients with CHB treated with PEG-IFNα for one year through the following recommendation levels: strong, > 50%; medium, 30–50%; weak, 10–30%; or no recommendation, < 10%.

### Statistical analysis and model establishment

Statistical analysis was conducted using SPSS software version 26.0 (SPSS, Inc, Chicago, Ill, USA). Quantitative parameters were presented using the mean ± standard deviation for normally distributed data, and the median (interquartile range) for non-normally distributed data. The data of continuous variables was compared using the Students’ *t*-test, Mann–Whitney *U* test, or Wilcoxon test wherever appropriate. Categorical variables were presented as counts and percentages and the comparisons were analyzed with χ^2^ test or Fisher’s exact test, wherever appropriate. Weighted kappa analysis was used to assess agreement between different time points. The cutoff values were determined using the receiver operating characteristic (ROC) curve, and the closest clinical applicable value to the cutoff value was considered as the optimal threshold for clinical convenience. The optimal threshold was used to classify the parameters; predictors of treatment outcomes were assessed through univariate and multivariate logistic regression analyses. All statistical analyses were two-sided at a significance level of 0.05. The predictive model was developed with the three best predictors from the logistic regression analysis using the stepwise method or enter method at baseline, and week 12 and 24. Scores of 1 or 3 were given if parameters met the optimal threshold values; otherwise, 0 was given. The scores were combined to compute the total scores.

## Results

### Baseline characteristics

Twenty-five patients were excluded: six had no baseline data, six missed follow-up before week 12, seven discontinued therapy during weeks 4–12 due to adverse events (three patients had symptoms of a severe flu, three had severe bone marrow suppression, and one had thyroid function abnormalities), five were examined by the Roche method, and one was excluded due to suspected data collection (Additional File 1: Figure S1). A total of 242 patients were enrolled in the final analysis. The response rates of patients receiving initial monotherapy, experienced monotherapy, initial combination, and experienced combination therapy were 31.78% (34/107), 40% (10/25), 26.67% (12/45) and 33.85% (22/65), respectively. Among them, 78 patients (32.2%) achieved HBsAg loss at the EOF, of whom 76 had serological conversion.

Responders were younger (34 vs. 38; P = 0.002) and had lower HBsAg (1.75 vs. 2.81; P < 0.001) and lower ALT (0.95 vs. 1.25; P = 0.013) at baseline than non-responders. There were no statistically significant differences in sex, HBV DNA, anti-HBc, treatment (initial vs. experienced treatment), and treatment protocol (PEG-IFNα monotherapy vs. combination therapy) and duration (all, P > 0.05) between the response and no-response groups (Table 1).

**Table 1:** Baseline characteristics of patients stratified by the loss of HBsAg at the follow-up endpoint

Characteristic	Total(n = 242)	RS(n = 78)	NRS(n = 164)	P value
Male, n (%)	197(81.40)	63(80.77)	134(81.71)	0.861
Age (years)	37(31–45)	34(30–41)	38(32–46)	**0.002**
ALT (×ULN)	1.15(0.80–1.80)	0.95(0.75–1.56)	1.25(0.86–1.88)	**0.013**
HBsAg (lg IU/ml)	2.28(1.60–3.11)	1.75(0.84–2.23)	2.81(2.01–3.30)	**P < 0.001**
Anti-HBc (S/CO)	9.45(8.57–10.54)	9.19(8.39–10.09)	9.53(8.64–10.63)	0.051
HBV DNA (lg copies/ml)	2.70(2.70–3.44)	2.70(2.70–3.08)	2.70(2.70–3.59)	0.069
Treatment modality				
Initial treatment (%)	152(62.81)	46(58.97)	106(64.63)	
Experienced treatment (%)	90(37.19)	32(41.03)	58(35.37)	0.395
Monotherapy (%)	132(54.55)	44(56.41)	88(53.66)	
Combination (%)	110(45.45)	34(43.59)	76(46.34)	0.688
Duration (months)	12.0 (6.0–12.0)	12.0 (6.0–12.0)	12.0 (8.0–14.0)	0.067

Data are expressed as number (%), mean ± standard deviation, or median (interquartile range). ALT, alanine aminotransferase; HBsAg, hepatitis B surface antigen; anti-HBc, antibody to hepatitis B core antigen HBV, NRS, non-responders; RS, responders; ULN, upper limit of normal

### Treatment and follow-up

**Fig. 1:**
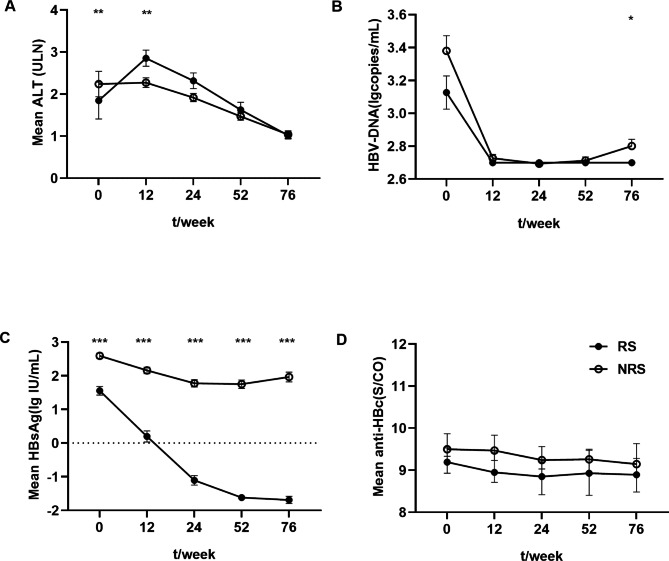
Kinetics of serum markers during PEG-IFNα therapy and follow-up between RS and NRS. (**A**) ALT. (**B**) HBV DNA. (**C**) HBsAg. (**D**) Anti-HBc. * P < 0.05, ** P < 0.01, and *** P < 0.001

ALT levels significantly increased in the response group at the beginning of treatment and peaked at 12 weeks, while no change was observed in the no-response group, posing a very significant difference (P < 0.01) (Fig. 1A). Serum HBV DNA notably declined at 12 weeks, at which most patients became negative in both groups. However, part of the no-response group became positive at EOF, resulting in significant differences between the two groups (P < 0.05) (Fig. 1B). HBsAg levels in the response group significantly and steadily decreased after treatment. However, HBsAg levels decreased only in the first 24 weeks, with an amplitude of < 1 Ig IU/mL, in the no-response group (all, P < 0.001) (Fig. 1C). Anti-HBc levels were relatively lower in responders during treatment and follow-up than in non-responders (all, P > 0.05) (Fig. 1D).

### Performance of HBsAg in predicting response

**Fig. 2:**
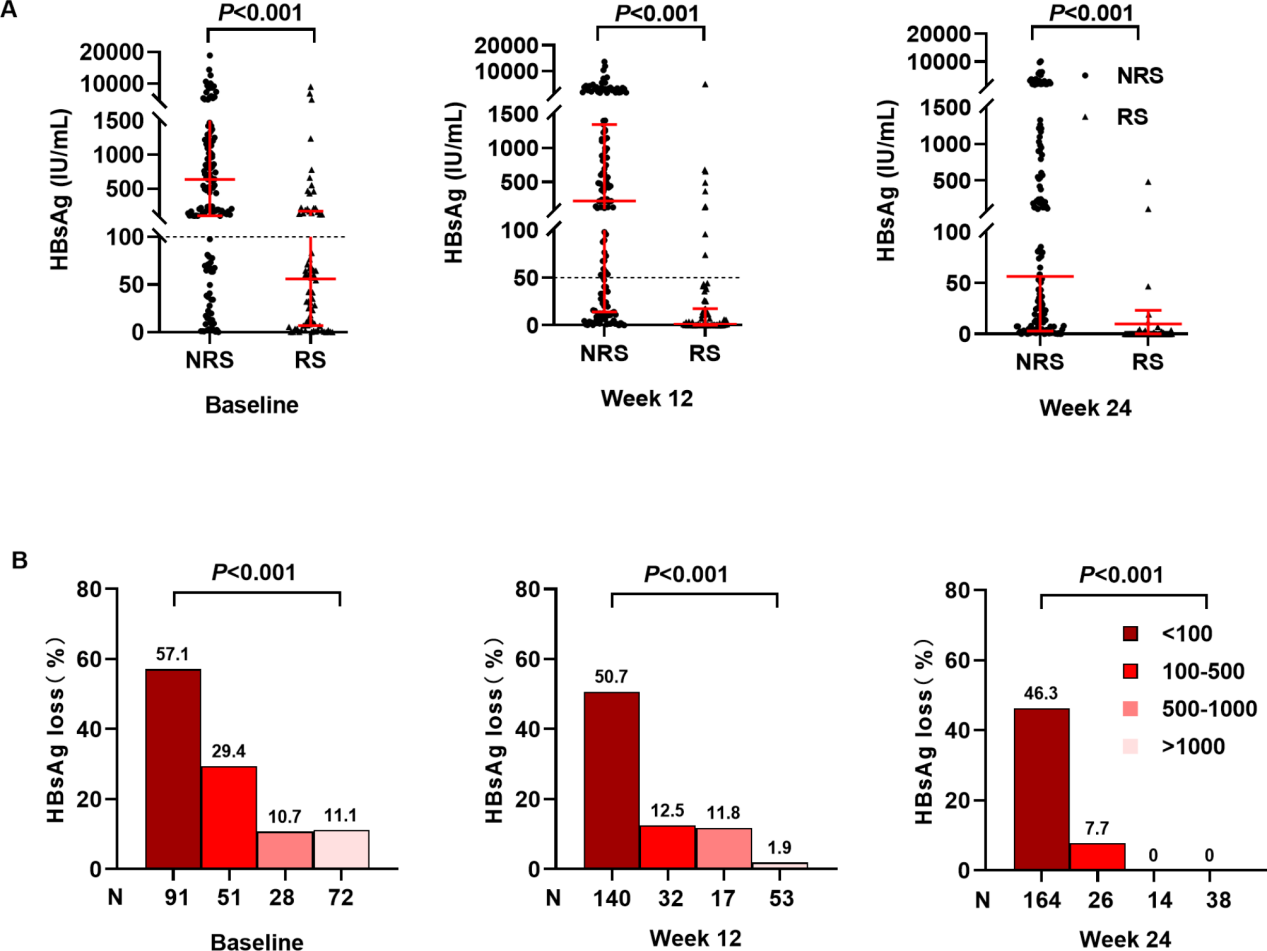
The performance of HBsAg in predicting response. (**A**) HBsAg distribution at different time points (baseline, week 12 and 24); (**B**) Rate of HBsAg loss at EOF was predicted based on HBsAg levels at different time points (baseline, week 12and 24)

HBsAg clearance was considered to indicate functional cure among patients with CHB. Therefore, baseline HBsAg was usually used as a predictor for treatment selection and prognosis prediction based on previous studies. Based on HBsAg distribution at baseline, and week 12 and 24, the response group had lower HBsAg levels than those of the no-response group (P < 0.001). It could still be found that patients with lower HBsAg levels failed to achieve functional cure, whereas those with higher HBsAg levels succeeded (Fig. 2A). HBsAg levels were classified into subgroups based on the following intervals: <100 IU/mL, 100–500 IU/mL, 500–1000 IU/mL, or > 1000 IU/mL at baseline, week 12, and 24. They were subsequently analyzed for response at EOF prediction. At baseline, the response rates for the different subgroups were 57.1%, 29.4%, 10.7%, and 11.1%, respectively. PEG-IFNα therapy was recommended to be administered weekly in patients with HBsAg > 100 IU/mL. Further, HBsAg clearance had little differences among those with HBsAg > 500 IU/mL. At week 12, 53 (21.9%) patients with HBsAg > 1000 IU/mL were able to stop PEG-IFNα treatment because of low response rates (1.9%), and at week 24, the response rates were 46.3%, 7.7%, and 0% for the HBsAg < 100 IU/mL, 100–500 IU/mL, and > 500 IU/mL groups, respectively (Fig. 2B).

### Performance of multiple parameters in predicting response

**Fig. 3:**
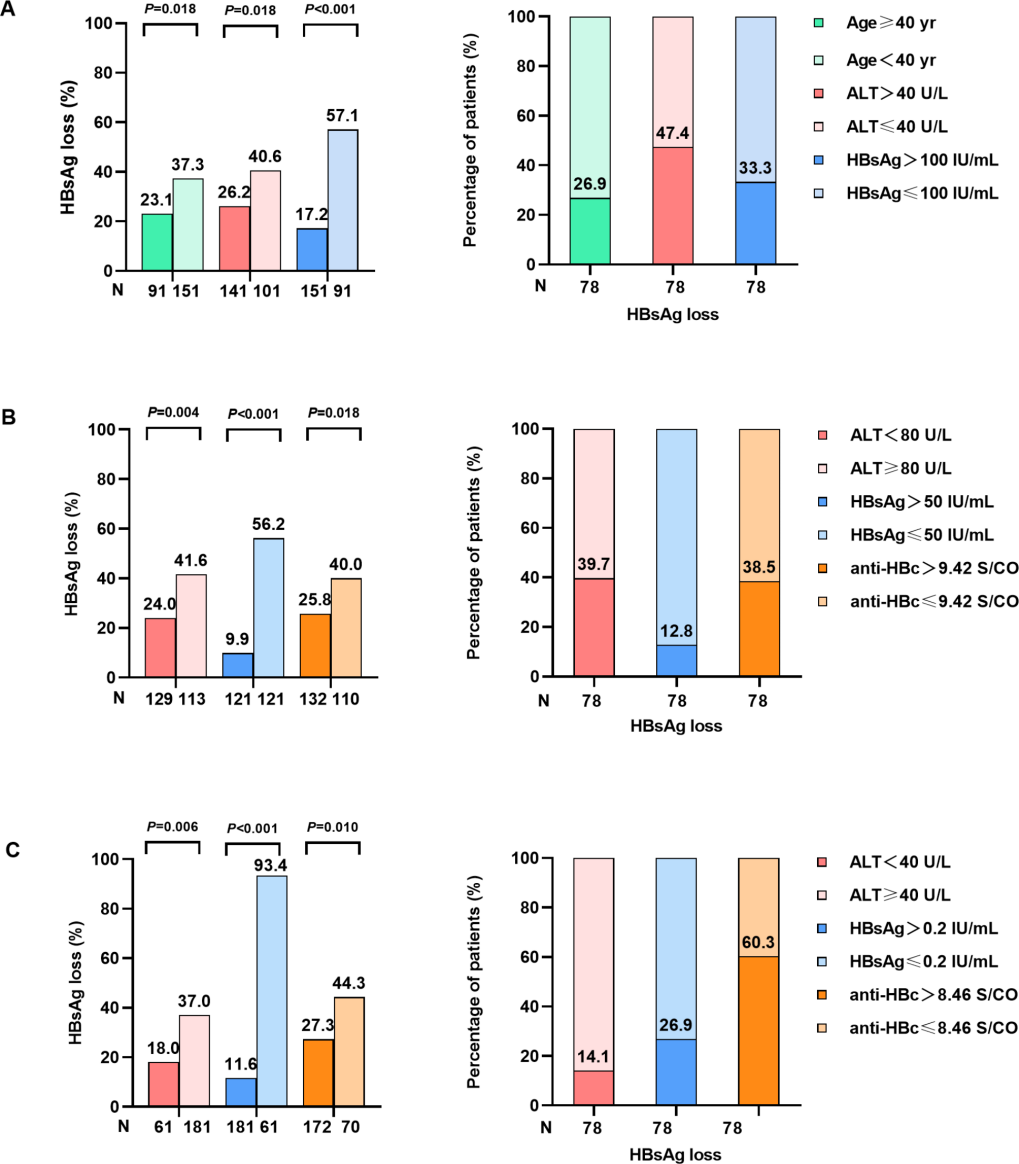
Predictive value of individual factors at different time points. (**A**) The HBsAg loss rate of three factors (Age, ALT, HBsAg) above and below the critical value and the proportion of each parameter in patients with loss of HBsAg at baseline. (**B**) HBsAg loss rate of three factors (ALT, HBsAg and anti-HBc) above and below the critical value and the proportion of each parameter among patients with loss of HBsAg at week 12. (**C**) HBsAg loss rate of three factors (ALT, HBsAg and anti-HBc) above and below the critical value and the proportion of each parameter among patients with loss of HBsAg at week 24

Clinically meaningful cutoff values were used to classify the parameters, and the best predictors of treatment outcomes were assessed through univariate and multivariate logistic regression analyses. The three best baseline predictors of response were age ≤ 40 years, ALT level ≤ 40 U/L, and HBsAg level ≤ 100 IU/mL (Additional File 2: Table S1). The differences in HBsAg seroclearance between groups were statistically significant (37.3% vs. 23.1; 40.6% vs. 26.2; and 57.1% vs. 17.2; all, P < 0.05); the values of relative risk (RR) were 1.61, 1.55, and 3.32, respectively. Furthermore, some patients with HBsAg loss were from the group with predicted poor curative effect (above cut-off values), with the response rates being 26.9%, 47.4%, and 33.3%, respectively (Fig. 3A).

At week 12, the three most meaningful predictors of response were an ALT level ≥ 80 U/L, HBsAg level ≤ 50 IU/mL, and anti-HBc level ≤ 8.42 S/CO (Table S1). These three variables were grouped according to clinically meaningful cut-off values, and the difference in HBsAg seroclearance was statistically significant (41.6% vs. 24.0%; 56.2% vs. 9.9%; and 40.0% vs. 25.8%; all, P < 0.05); the values of RR were 1.73, 5.68, and 1.55, respectively. Furthermore, 12.8–39.7% of patients with HBsAg loss were from the group with predicted poor curative effect (Fig. 3B).

At week 24, the three most significant predictors of response were an ALT level ≥ 40 U/L (P < 0.006), HBsAg level ≤ 0.2 IU/ml (P < 0.001), and anti-HBc level ≤ 8.46 S/CO (P = 0.010) (Additional File 1: Table S1). These three variables were grouped according to clinically meaningful cut-off values, and the difference in HBsAg seroclearance between groups was statistically significant (37.0% vs. 18.0%, 93.4% vs. 11.6%, and 44.3% vs. 27.3%; all, P < 0.05); the values of RR were 2.06, 8.05 and 1.62, respectively. Furthermore, 14.1–60.3% of patients with HBsAg loss were from the group with predicted poor curative effect. The predictive value of anti-HBc was the lowest (Fig. 3C).

### Multi-parameter score model in predicting response

**Fig. 4:**
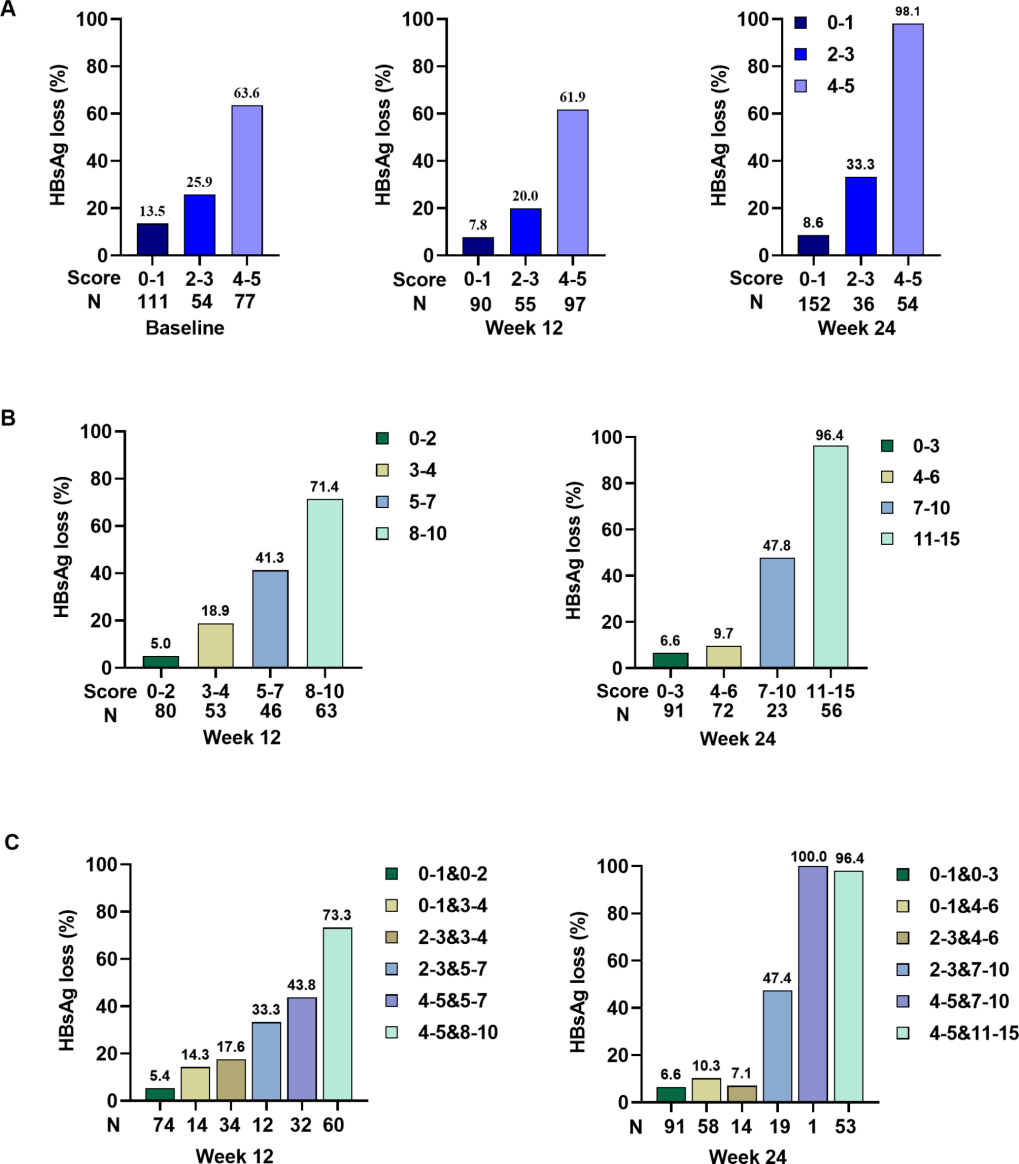
Integral or/and cumulative total scores combined at each time point that predicted the loss of HBsAg. (**A**) Integral score at baseline, week 12 and 24 week. (**B**) Cumulative total scores at week 12 and 24. (**C**) Integral and cumulative total scores at week 12 and 24

The corresponding integrals were endowed according to the various odds ratio (OR) values of predictor variables. A score of 1 was given if patients had a change in each of the selected predictive parameters, and a score of 3 was given if the OR value of the factor studied was more than two times higher than that of the remaining values. Accordingly, the best three predictors of response at baseline were found to be age ≤ 40 years, an ALT level ≤ 40 U/L, and an HBsAg level ≤ 100 IU/mL; the OR values were 2.08, 2.28, and 7.89, respectively (Additional File 2: Table S1). They were respectively integrated with 1, 1, and 3 points when the three parameters reached the optimal threshold (Table 2). The response rates of patients with scores ranging from 0 to 5 were 17.6%, 11.7%, 27.5%, 21.4%, 59.2%, and 71.4%, respectively. The HBsAg loss rates of the group that scored 2 (27.5%) were even higher than that of the group that scored 3 (21.4%), suggesting that, although the single predictive value of age or ALT levels was inferior to that of HBsAg levels in terms of individual factors, combining these two factors significantly enhanced the prediction’s efficiency (Additional File 3: Figure S2 A). The scores were combined for the convenience of clinical application. For patients with scores of 0–1, 2–3, and 4–5, the response rates were 13.5% (15/111), 25.9% (14/54), and 63.6% (49/77), respectively (Fig. 4A).

**Table 2:** The factors most related to the loss rate of HBsAg and their corresponding scoring values

	Factors	Score
Baseline	Age ≤ 40 yr	1
	ALT ≤ 40 U/L	1
	HBsAg ≤ 100 IU/mL	3
12 W	ALT ≥ 80 U/L	1
	anti-HBc ≤ 9.42 S/CO	1
	HBsAg ≤ 50 IU/mL	3
24 W	ALT ≥ 40 U/L	1
	anti-HBc ≤ 8.46 S/CO	1
	HBsAg ≤ 0.2 IU/mL	3

ALT, Alanine aminotransferase; HBsAg, hepatitis B s antigen; anti-HBc, antibody to hepatitis B core antigen; w., week

At week 12, the best three predictors of response were an ALT level ≥ 80 U/L, anti-HBc level ≤ 8.42 S/CO, and HBsAg level ≤ 50 IU/mL; the OR values were 2.17, 2.30, and 17.48, respectively (Additional File 2: Table S1). They were respectively integrated with 1, 1, and 3 points when the three parameters reached the optimal threshold (Table 2). The response rates of patients with scores of 0, 1, 2, 3, 4, and 5 were 3.6%, 14.3%, 9.7%, 33.3%, 57.1%, and 74.1%, respectively (Additional File 3: Figure S2 A). The scores were combined for the convenience of clinical application. For patients with scores of 0–1, 2–3, and 4–5, the response rates were 7.8% (7/90), 20.0% (11/55) and 61.9% (60/97), respectively (Fig. 4A).

At week 24, the best three predictors of response were an ALT level ≥ 40 U/L, anti-HBc level ≤ 8.46 S/CO, and HBsAg level ≤ 0.2 IU/mL; the OR values were 20.17, 3.69, and 501.66, respectively (Additional File 2: Table S1). They were respectively integrated with 1, 1, and 3 points when the three parameters reached the optimal threshold (Table 2). The response rates of patients with scores of 0, 1, 2, 3, 4, and 5 were 10.3%, 9.8%, 26.7%, 66.7% and 97.4%, respectively (Additional File 3: Figure S2 A). The scores were combined for the convenience of clinical application. For patients with scores of 0–1, 2–3, and 4–5, the response rates were 8.6% (13/152), 33.3% (12/36), and 98.1% (53/54), respectively (Fig. 4A).

### Prediction and evaluation based on RGT strategy

**Fig. 5:**
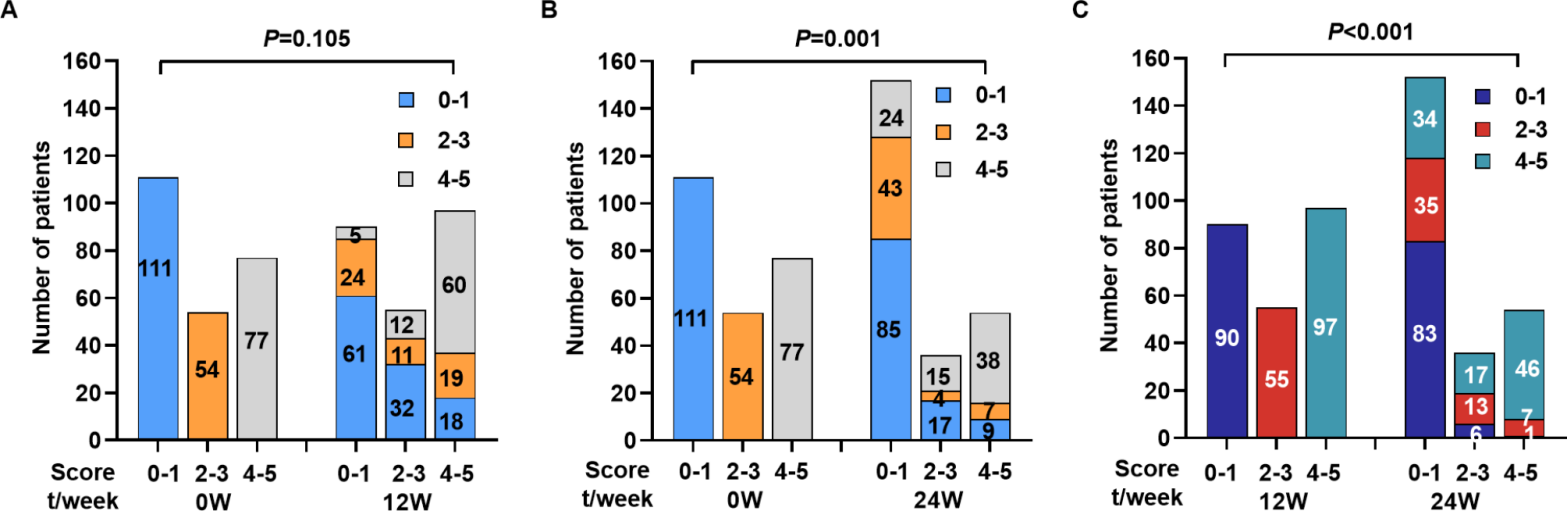
Comparison of the changes in patients with each type of score at different time points. (**A**) Baseline compared with week 12. (**B**) Baseline compared with weeks 24. (**C**) Week 12 compared with week 24

Patients with low scores had a lower response rate at the different time points (Fig. 4A). However, the scores of the same patient may have significantly differed between various time points (Fig. 5). At week 12, 32 (28.8%) and 18 (16.2%) patients who scored 0–1 at baseline had improved scores of 2–3 and 4–5, respectively. Among the patients who scored 2–3 at baseline, 44.4% (24/54) had decreased scores of 1–2, and 35.2% (19/54) had improved scores of 4–5. Only 22.1% (17/77) of patients who scored 4–5 at baseline had a score change (Fig. 5A). Analysis of the interrater agreement showed a kappa value of 0.302 (P < 0.001) (Additional File 4 and 5: Table S2, S3).

At week 24, 17 (15.3%) and 9 (8.1%) patients who scored 0–1 at baseline had improved scores of 2–3 and 4–5, respectively. Among the patients who scored 2–3 at baseline, 79.6% (43/54) had decreased scores of 0–1 point, and 13.0% (7/54) had improved scores of 4–5 points. In total, 31.2% (24/77) and 19.5% (15/77) of patients who scored 4–5 at baseline had decreased scores of 0–1 and 2–3 points, respectively (Fig. 5B). Analysis of the interrater agreement showed a kappa value of 0.221 (P < 0.001) (Additional File 4 and 6: Table S2, S4).

At week 12, the total number of patients whose scores are 0–1, 2–3 and 4–5 was 90, 55 and 97, respectively. In total, 7.8% (7/90) of patients who scored 0–1 at week 12 had a score change at week 24. Of the patients who scored 2–3 at week 12, 63.6% (35/55) converted to 0–1 point, and 12.7% (7/55) converted to 4–5 points. At week 24, 35.1% (34/97) and 17.5% (17/97) of patients who scored 4–5 points at week 12 had scores of 0–1 and 2–3, respectively (Fig. 5C). Analysis of the interrater agreement showed a kappa value of 0.361 (P < 0.001) (Additional File 4 and 7: Table S2, S5).

The total score at week 12 was obtained by adding each patient’s score from baseline to week 12, and the cumulative score range was 0–10 points. It was found that the efficacy significantly increased with the total score (Additional File 3: Figure S2 B). At week 12, patients with scores of 0–1 and/or cumulative score of 0–2 had effective rates of 5.4% and 7.3%, respectively; patients with scores of 2–3 and/or cumulative score of 3–4 had effective rates of 17.6% and 20.3%, respectively; patients with scores of 4–5 and/or cumulative score of 5–7 had effective rates of 43.8% and 58.6%, respectively. For patients with scores of 4–5 and/or cumulative score of 8–10, efficacy was 73.3% and 61.0%, respectively (Fig. 4B, C).

The total score at week 24 was obtained by adding each patient’s score from baseline to week 24, and the cumulative score range was 0–15 points (Additional File 3: Figure S2 B). The effective rates of patients with scores of 0–1 and/or cumulative score of 0–3 total were 6.6% and 8.6%; patients with scores of 2–3 and/or cumulative score of 4–6 had effective rates of 7.1% and 19.1%, respectively; patients with scores of 2–3 and/or cumulative score of 7–10 had effective rates of 47.4% and 35.0%, respectively. For patients with scores of 4–5 and/or cumulative score of 11–15, efficacy was 96.4% and 96.4%, respectively (Fig. 4B, C).

### Results obtained from the model we explored

**Fig. 6:**
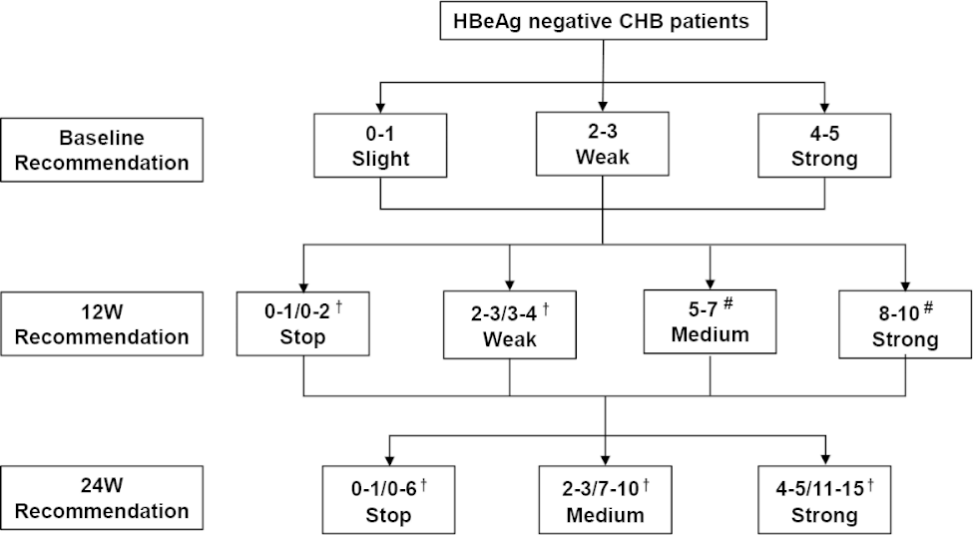
Flow chart of the clinical evaluation recommendations based on a RGT strategy. † refers to the scoring or accumulating total scores at each point in time. # refers to the accumulating total scores at each point in time

According to the patients’ response to treatment at weeks 12 and/or 24, the integral and cumulative integral model were used to predict the possible response rates and timely make the decision to stop or continue PEG-IFNα therapy (Fig. 6). At baseline, for HBeAg-negative CHB patients with scores of 0–1, 2–3, or 4–5, our recommendations for PEG-IFN use were slight, weak, and strong, respectively. At week 12, for patients with 0–1 score or 0–2 cumulative score, it is recommended to stop treatment directly; PEG-IFN is weakly recommended for patients with a score of 2–3 or a cumulative score of 3–4; PEG-IFN therapy is moderately or strongly recommended for patients with a cumulative score of 5–7 or 8–10.

At week 24, for patients with 0–1 score or 0–6 cumulative score, we recommend stopping treatment; for patients with scores of 2–3 or cumulative scores of 7–10, we give moderate recommendations; for patients with a score of 4–5 or an overall score of 11–15 we strongly recommend continued treatment.

## Discussion

PEG-IFNα monotherapy or combined NUCs therapy are often used for HBeAg**-**negative patients with CHB to achieve functional cure, but their efficacy is still very limited. Many scholars used predictive parameters such as HBsAg, HBV-DNA, and viral genotypes to establish some prediction models. Particularly, it was found that patients with an HBsAg level < 1500 IU/mL were more likely to have HBeAg seroclearance [35–39]. However, these models typically were single center-based, focused on single parameters at baseline, and/or had a small sample size. The predictive efficacy was low, and the reference value for clinical application was limited. Data for the present study were collected from 2 hospitals. Patients were divided into the response and non-response groups according to whether HBsAg clearance occurred at EOF. The three most valuable predictors were determined to build the predictive model by univariate and multivariate analyses using logistic regression at different time points.

Similar to previous studies, this study found that HBsAg was the most predictive parameter at each time point. Moreover, the predictive value of HBsAg was found to have increased with treatment time [40, 41]. For example, patients with HBsAg at a level of > 1000 or 500 IU/mL at weeks 12 and 24 were rarely able to achieve effective response, and the percentage of such patients was only 20% approximately. Results also demonstrated that many responders had higher HBsAg levels early on that decreased rapidly after treatment, reaching HBsAg clearance or serological conversion at EOF. It was found that single parameters, such as age and ALT levels, at baseline were of limited value for predicting HBsAg loss, but the response rates of patients who scored 2 (age and ALT) or 3 (HBsAg only) were 27.5% and 21.4%, respectively. In patients with scores ≥ 4 (HBsAg, age and/or ALT) the response rates were 59.2–71.4%, indicating that the combined model had a remarkably improved prediction efficiency and far better accuracy than the single-factored HBsAg prediction model. Predictors obtained at different time points have similar effects when used alone or in combination. Although the HBsAg levels have a good predictive value, the combined model is still superior.

The predictive value of the combination model established at baseline, and weeks 12 and 24 can distinguish well which patients are suitable for PEG-IFNα therapy. However, there were significant differences in scores at different time points, and the kappa value was only 0.221–0.361, indicating that the predictions may have differed between time points and that errors may have occurred. In total, 13.5% of patients who scored 0–1 at baseline responded to treatment, indicating a potential therapeutic value. When the score was 0–1 at week 12 and 24, the response rates dropped to 7.8% and 8.6%, respectively, and the therapeutic value decreased significantly. Moreover, the combined factors, including HBsAg, ALT, and anti-HBC levels, at week 12 and 24 were closely related to the therapeutic response and mechanism of PEG-IFNα. Therefore, an RGT-based strategy that comprehensively determines whether PEG-IFNα therapy should be stopped according to the patient’s response to therapy can be adopted.

It was very important to make predictions more accurate based on cumulative integrals due to the poor consistency of integrals at different time points. Therefore, this study comprehensively considered the combined application of prediction models at different time points, introducing cumulative integrals. Cumulative integrals at week 12 and 24 had a good complementary role in predicting the response rates at these time points. For example, for patients with a score of 2–3 at week 24 and a cumulative total score of 4–6 and 7–10, the response rates were 7.1% and 47.4%, respectively. It was further indicated that an RGT strategy could improve the accuracy of prediction and help patients and physicians decide whether the treatment plan currently used should be adjusted.

Previously, Hu et al. [42]. and Qin et al. [43]. established a prediction model for the clinical cure of patients with CHB treated with PEG-IFNα based on quantitative HBsAg values. They also developed related strategies using a combination of baseline-guided therapy and RGT. However, these schemes only focused on the influence of a single time point or a single parameter of HBsAg on the prediction effect. As shown in this study, HBsAg at each time point had a good reference value for predicting HBsAg loss at EOF. Moreover, other single indicators such as age, and ALT and anti-HBc levels also had predictive value. However, compared with HBsAg, the predictive value of a single indicator was limited, and the combined application of related indicators could achieve a similar effect as that of HBsAg. Therefore, the comprehensive model was obviously better than the single-factored HBsAg prediction model. Our team believed that the baseline combined indicator prediction model could be used to make preliminary classification of patients, allowing them to decide whether to choose PEG-IFNα therapy. According to the patients’ response to treatment at week 12 and/or 24, the integral and cumulative integral model were used to predict the possible response rates and timely make the decision to stop or continue PEG-IFNα therapy (Fig. 6). Moreover, there was no significant difference in the response rates of initially treated or experienced patients, despite differences in their baseline data. The same results were seen for patients undergoing monotherapy or combination therapy. This model was used to classify and analyze the data of each group. It was found that there were significant differences in the response rates of different integrals within each group, but the differences between groups were very limited. It is further indicated that this model based on an RGT strategy could be applied to all HBeAg-negative patients with CHB treated with PEG-IFNα (Additional File 8: Figure S3-6). However, this study seemed to overthrow the previous New-SWITCH [42] and OSST [43] studies from China because in the current study, the combination or sequential use of Peg-IFN with NA is not a factor for HBsAg loss at EOF. The results of our study differ from previous RCTs because it is a retrospective and not a prospective study. The prospective studies were more homogeneous at enrollment and standardized in medication. The results presented in the article are reflected by the whole data. Nonetheless, this study was carried out only in two centers, and the sample size was relatively limited. In addition, our study shows that native patients treated with combination therapy had a lower HBsAg clearance rate than those treated with PEG-IFN monotherapy. Before choosing treatment strategies, it is necessary to evaluate the indexes related to efficacy and safety. Patients were recommended to receive combination therapy at baseline in HBeAg-negative with higher HBsAg levels and a higher viral load, leading to a lower clearance rate. Moreover, in our study, patients adding on PEG-IFN therapy and switching from NUCs to PEG-IFN therapy achieved functional cure easier than native patients only treated with PEG-IFN therapy, which is similar to a previous study [44]. However, the difference was not statistically significant.

The team made different recommendations based on the opinions of experts and patients, as well as a comprehensive evaluation of treatment response rate, drug side effects, health economics, advantages and disadvantages of alternative treatment, and patients’ mental panic induced by the disease (discrimination and liver cancer). This model is expected to yield maximum benefit with minimum pay. However, in the process of clinical diagnosis and treatment, it is also necessary to make a comprehensive evaluation and decision according to the individual situation of each patient. For example, patients with serious side effects and an expected response rate of 10–30% can consider stopping treatment, whereas individuals with a strong willingness to treat and no obvious side effects can consider continuing treatment.

We established the prediction models for PEG-IFNα response in HBeAg-negative patients with CHB achieving functional cure and developed a clinically practical treatment decision-making process based on a RGT strategy. Surely, as this study is only from two centers, and the sample size was relatively limited, further large-scale multi-center studies to confirm the results presented herein are warranted. Moreover, there may be differences in the response rates among different populations or patients receiving different treatment schemes; hence, whether independent models need to be established warrants further exploration.

## Conclusion

In summary, our study successfully established a multi-parameter prediction model for the functional cure of HBeAg-negative patients with CHB treated with PEG-IFNα. The three most meaningful predictors were age ≤ 40 years, ALT levels ≤ 40 U/L, and HBsAg levels ≤ 100 IU/mL at baseline; ALT levels ≥ 80 U/L, anti-HBc levels ≤ 8.42 S/CO, and HBsAg levels ≤ 50 IU/mL at week 12; and ALT levels ≥ 40 U/L, anti-HBc levels ≤ 8.46 S/CO, and HBsAg levels ≤ 0.2 IU/mL at week 24. For HBeAg-negative patients meeting different dominance factors at different times, the higher the score and/or cumulative score, the more PEG-IFN therapy is strongly recommended. For patients with a score of 0–1 or cumulative scores of 0–2 at 12 weeks and for those with a score of 0–1 or cumulative scores of 0–6 at 24 weeks are recommended to stop PEG-IFN treatment. The prediction model is simplistic and practical, and the RGT strategy can help to optimize the use of PEG-IFNα.

## Electronic supplementary material

Below is the link to the electronic supplementary material.

**Figure S1** Flow diagram for the enrolment and exclusion of patients.

**Table S1** Univariate and multivariate analysis of factors associated with sustained off-treatment virological response.

**Figure S2** The integral or cumulative total score uncombined predicted the loss rate of HBsAg at EOF. (A) Integral score at baseline,week 12.and 24. (B) Cumulative total score at week 12.and 24

**Table S2** Consistency analysis of integration at different time points

**Table S3** Weighted Kappa consistency test between baseline and 12W.

**Table S4** Weighted Kappa consistency test between baseline and 24W

**Table S5** Weighted Kappa consistency test between 12W and 24W

**Figure S3** Application of the score model in the initial treatment of patients with PEG-IFN? monotherapy.

## Data Availability

The datasets used and/or analyzed during the current study available from the corresponding author on reasonable request.

## List of abbreviations

RGT: Response-guided therapy
CHB: Chronic hepatitis B
PEG-IFNα: Pegylated interferonα
EOF: End of follow-up
HBsAg: Hepatitis B surface antigen
ALT: Alanine aminotransferase
NUCs: Nucleoside analogs
RR: Relative risk
OR: Odds ratio
NRS: Non-responders
RS: Responders
Anti-HBc: Antibody to hepatitis B core antigen
