# Supplementary material for: Establishment of a multi-parameter prediction model for the functional cure of HBeAg-negative chronic hepatitis B patients treated with pegylated interferonα and decision process based on response-guided therapy strategy
Source: BMC Infect Dis. 2023 Jul 10;23:456. doi: 10.1186/s12879-023-08443-1 (PMC10332036; doi:10.1186/s12879-023-08443-1)
Supplement: Supplementary file 1 — Figure S1 Flow diagram for the enrolment and exclusion of patients. [file 12879_2023_8443_MOESM1_ESM.docx]

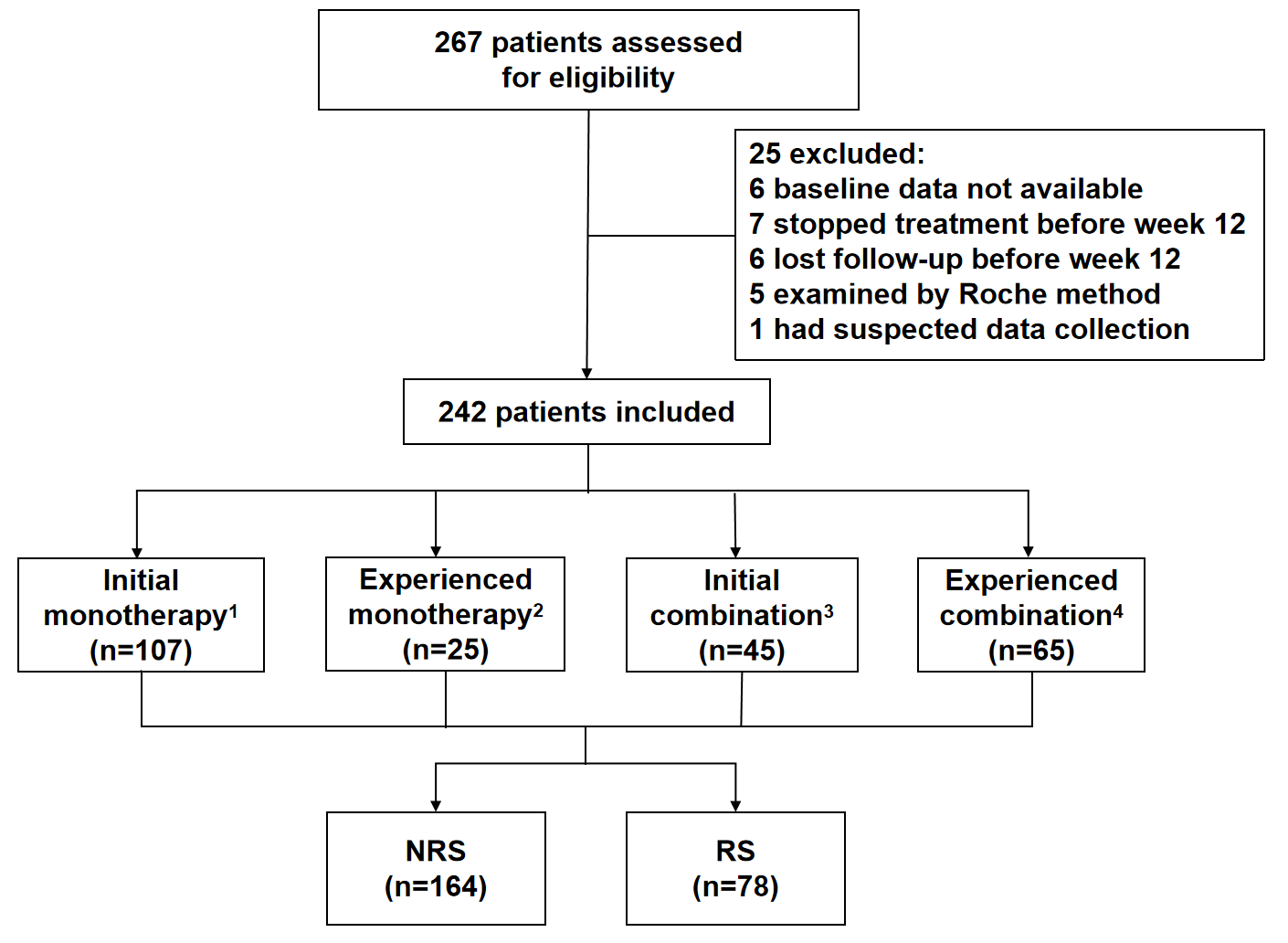


**Figure S1** Flow diagram for the enrolment and exclusion of patients.

1 Only PEG-IFN therapy.

2 Switching from NUCs to PEG-IFN therapy.

3 PEG-IFN combined with NUCs therapy.

4 NUCs adding on PEG-IFN therapy.
