# Supplementary material for: Establishment of a multi-parameter prediction model for the functional cure of HBeAg-negative chronic hepatitis B patients treated with pegylated interferonα and decision process based on response-guided therapy strategy
Source: BMC Infect Dis. 2023 Jul 10;23:456. doi: 10.1186/s12879-023-08443-1 (PMC10332036; doi:10.1186/s12879-023-08443-1)
Supplement: Supplementary file 2 — Table S1 Univariate and multivariate analysis of factors associated with sustained off-treatment virological response. [file 12879_2023_8443_MOESM2_ESM.docx]

**Table S1** Univariate and multivariate analysis of factors associated with sustained off-treatment virological response**.**

|  | Selected predictive variables | Univariate analysis OR (95% CI) | P Value | Multivariate analysis OR (95% CI) | P Value |
| --- | --- | --- | --- | --- | --- |
| Baseline | Age≤40 (yr) | 2.41(1.23-4.74) | **0.019** | 2.08(1.03-4.19) | **0.04** |
|  | Sex (male) | 1.06(0.53-2.12) | 0.861 |  |  |
|  | ALT≤40 U/L | 2.34(1.35-4.08) | **0.019** | 2.28(1.19-4.34) | **0.003** |
|  | HBsAg≤100 IU/mL | 6.962(3.83-12.66) | **P<0.001** | 7.89(3.80-16.39) | **P<0.001** |
|  | Anti-HBc≤10.12 S/CO | 2.18(1.17-4.07) | **0.014** |  |  |
|  | HBV DNA≤500 copies/mL | 1.86(1.04-3.34) | **0.037** |  |  |
|  | Treatment modality |  |  |  |  |
|  | Initial treatment | 1.27(0.73-2.21) | 0.395 |  |  |
|  | Combination | 0.90(0.52-1.54) | 0.688 |  |  |
| 12W | Age≤40 (yr) | 2.41(1.23-4.74) | **0.019** |  |  |
|  | ALT≥80 U/L | 2.285(1.29-4.05) | **0.006** | 2.17(1.08-4.39) | **0.031** |
|  | HBsAg≤50 IU/mL | 14.51(6.67-31.60) | **P<0.001** | 17.48(7.50-40.63) | **P<0.001** |
|  | Anti-HBc≤9.42 S/CO | 1.92(1.09-3.39) | **0.025** | 2.30(1.15-4.63) | **0.019** |
| 24W | Age≤40 (yr) | 2.41(1.23-4.74) | **0.019** |  |  |
|  | ALT＞40 U/L | 4.447(1.50-13.18) | **0.007** | 20.17(1.78-228.36) | **0.015** |
|  | HBsAg≤0.20 IU/mL | 142.59(40.39-503.14) | **P<0.001** | 501.66(60.86-4135.00) | **P<0.001** |
|  | Anti-HBc≤8.46 S/CO | 1.98(1.08-3.64) | **0.028** | 3.69(1.31-10.35) | **0.013** |
| 52W | Age≤40 (yr) | 2.41(1.23-4.74) | **0.019** |  |  |
|  | ALT>100 U/L | 2.38(0.91-6.16) | **0.069** | 2.77(0.43-17.86) | 0.285 |
|  | HBsAg≤0.20 IU/mL | 221.34(67.50-725.80) | **P<0.001** | 271.45(74.04-955.19) | **P<0.001** |
|  | Anti-HBc≤7.83 S/CO | 2.43(1.18-5.03) | **0.016** | 1.13(0.24-5.43) | 0.877 |

The enter method in logistic regression analysis instead of stepwise logistic regression analysis is used. OR, Odds ratio; CI, confidence interval ; ALT, Alanine aminotransferase; HBsAg, hepatitis B s antigen; anti-HBc, antibody to hepatitis B core antigen; HBV, hepatitis B virus; ULN, upper limit of normal; w, week；yr, year.
