# Supplementary material for: Establishment of a multi-parameter prediction model for the functional cure of HBeAg-negative chronic hepatitis B patients treated with pegylated interferonα and decision process based on response-guided therapy strategy
Source: BMC Infect Dis. 2023 Jul 10;23:456. doi: 10.1186/s12879-023-08443-1 (PMC10332036; doi:10.1186/s12879-023-08443-1)
Supplement: Supplementary file 3 — Figure S2 The integral or cumulative total score uncombined predicted the loss rate of HBsAg at EOF. (A) Integral score at baseline,week 12.and 24. (B) Cumulative total score at week 12.and 24 [file 12879_2023_8443_MOESM3_ESM.docx]

**
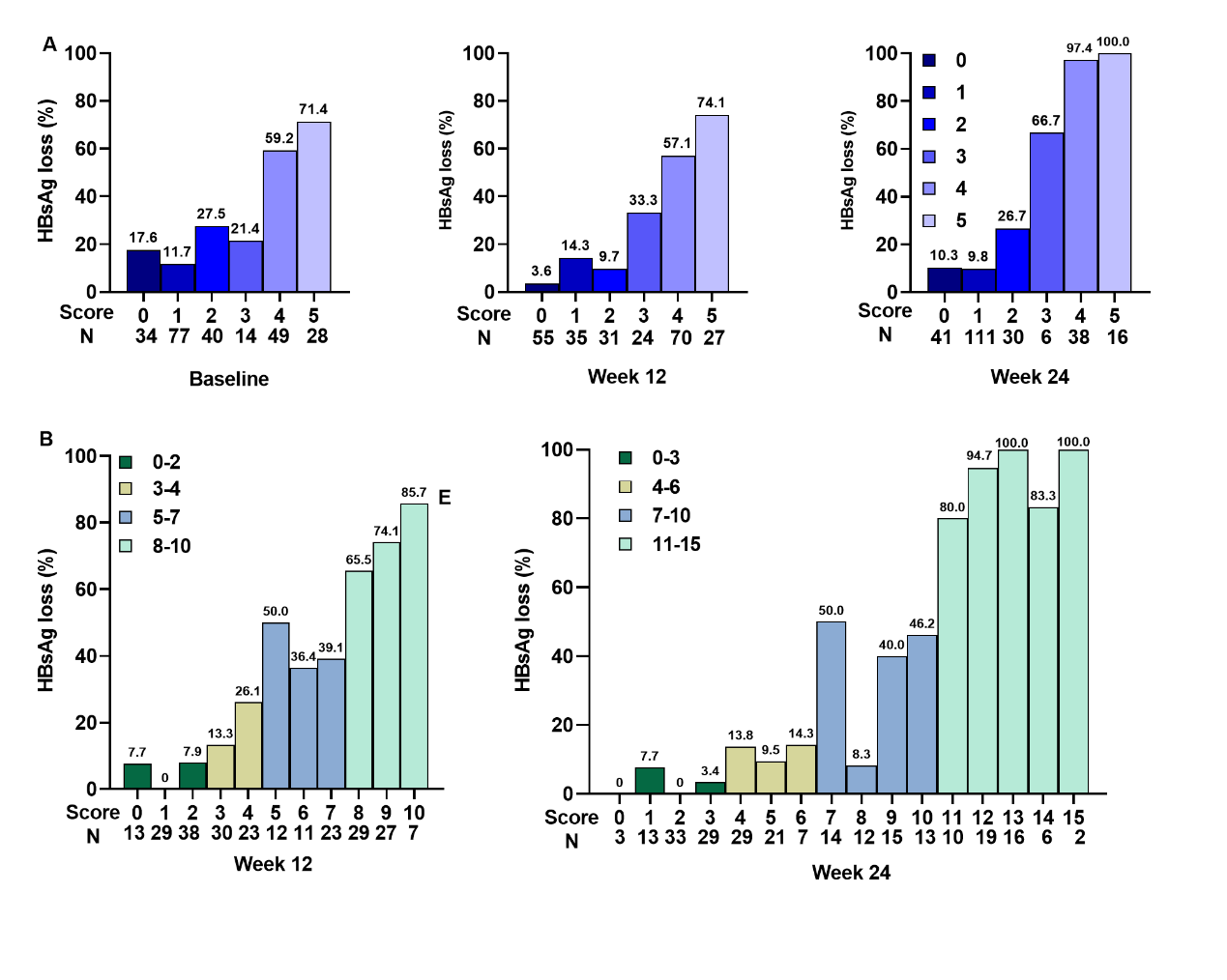
**

**Figure S2** The integral or cumulative total score uncombined predicted the loss rate of HBsAg at EOF. **(A)** Integral score at baseline,week 12.and 24. **(B)** Cumulative total score at week 12.and 24
