# Supplementary material for: Establishment of a multi-parameter prediction model for the functional cure of HBeAg-negative chronic hepatitis B patients treated with pegylated interferonα and decision process based on response-guided therapy strategy
Source: BMC Infect Dis. 2023 Jul 10;23:456. doi: 10.1186/s12879-023-08443-1 (PMC10332036; doi:10.1186/s12879-023-08443-1)
Supplement: Supplementary file 4 — Table S2 Consistency analysis of integration at different time points [file 12879_2023_8443_MOESM4_ESM.docx]

**Table S2** Consistency analysis of integration at different time points

|  | Kappa coefficient | *P* value |
| --- | --- | --- |
| Baseline VS 12W | 0.302 | ＜0.001 |
| Baseline VS 24W | 0.221 | ＜0.001 |
| 12W VS 24W | 0.361 | ＜0.001 |
