# Supplementary material for: Establishment of a multi-parameter prediction model for the functional cure of HBeAg-negative chronic hepatitis B patients treated with pegylated interferonα and decision process based on response-guided therapy strategy
Source: BMC Infect Dis. 2023 Jul 10;23:456. doi: 10.1186/s12879-023-08443-1 (PMC10332036; doi:10.1186/s12879-023-08443-1)
Supplement: Supplementary file 6 — Table S4 Weighted Kappa consistency test between baseline and 24W [file 12879_2023_8443_MOESM6_ESM.docx]

**Table S4** Weighted Kappa consistency test between baseline and 24W

| Time |  | 24W | | | |
| --- | --- | --- | --- | --- | --- |
|  | Score | 0-1 | 2-3 | 4-5 | Total |
| Baseline | 0-1 | 85(3) | 17(3) | 9(9) | 111(15) |
|  | 2-3 | 43(5) | 4(2) | 7(7) | 54(14) |
|  | 4-5 | 24(5) | 15(7) | 38(37) | 77(49) |
|  | Total | 152(13) | 36(12) | 54(53) | 242(78） |

Note: The figures in parentheses represent the number of patients with HBsAg loss at EOF
