# Supplementary material for: Establishment of a multi-parameter prediction model for the functional cure of HBeAg-negative chronic hepatitis B patients treated with pegylated interferonα and decision process based on response-guided therapy strategy
Source: BMC Infect Dis. 2023 Jul 10;23:456. doi: 10.1186/s12879-023-08443-1 (PMC10332036; doi:10.1186/s12879-023-08443-1)
Supplement: Supplementary file 7 — Table S5 Weighted Kappa consistency test between 12W and 24W [file 12879_2023_8443_MOESM7_ESM.docx]

**Table S5** Weighted Kappa consistency test between 12W and 24W

| Time |  | 24W | | | |
| --- | --- | --- | --- | --- | --- |
|  | Score | 0-1 | 2-3 | 4-5 | Total |
| 12W | 0-1 | 83(5) | 6(1) | 1(1) | 90(7) |
|  | 2-3 | 35(2) | 13(2) | 7(7) | 55(11) |
|  | 4-5 | 34(6) | 17(9) | 46(45) | 97(60) |
|  | Total | 152(13) | 36(12) | 54(53) | 242(78) |

Note: The figures in parentheses represent the number of patients with HBsAg loss at EOF
