# Supplementary material for: Establishment of a multi-parameter prediction model for the functional cure of HBeAg-negative chronic hepatitis B patients treated with pegylated interferonα and decision process based on response-guided therapy strategy
Source: BMC Infect Dis. 2023 Jul 10;23:456. doi: 10.1186/s12879-023-08443-1 (PMC10332036; doi:10.1186/s12879-023-08443-1)
Supplement: Supplementary file 8 — Figure S3 Application of the score model in the initial treatment of patients with PEG-IFN? monotherapy. [file 12879_2023_8443_MOESM8_ESM.docx]

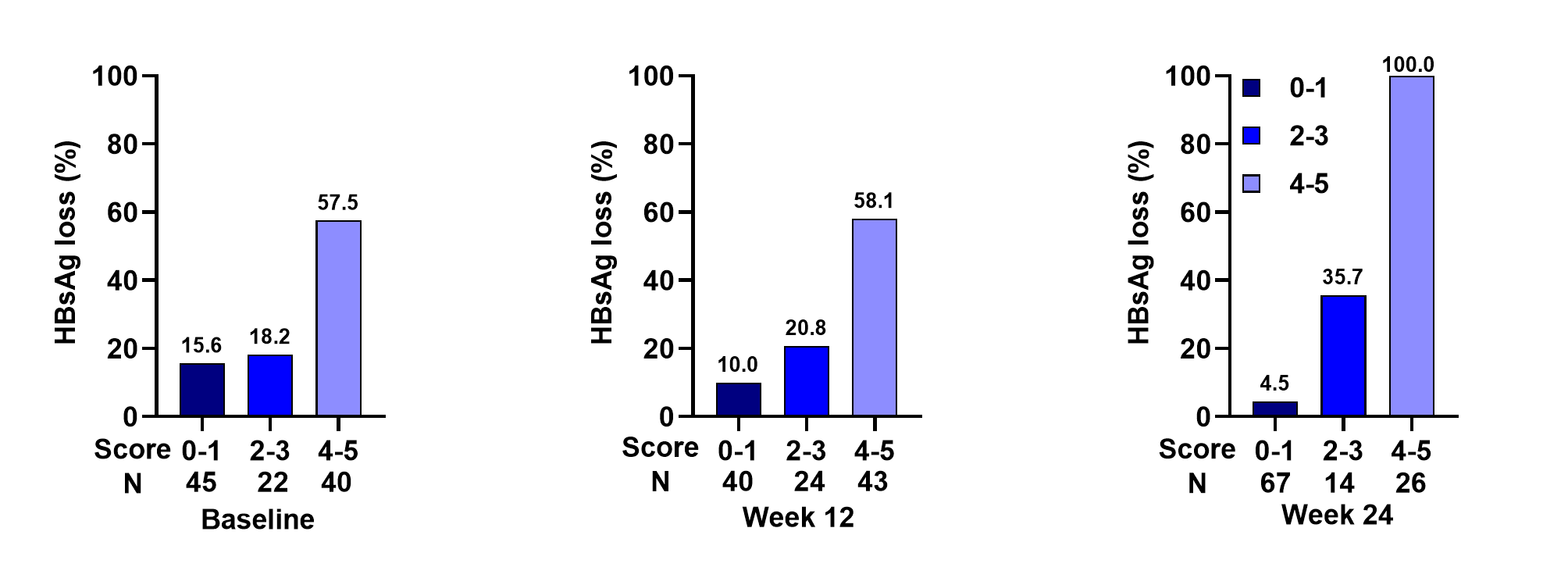


**Figure S3** Application of the score model in the initial treatment of patients with PEG-IFNα monotherapy.


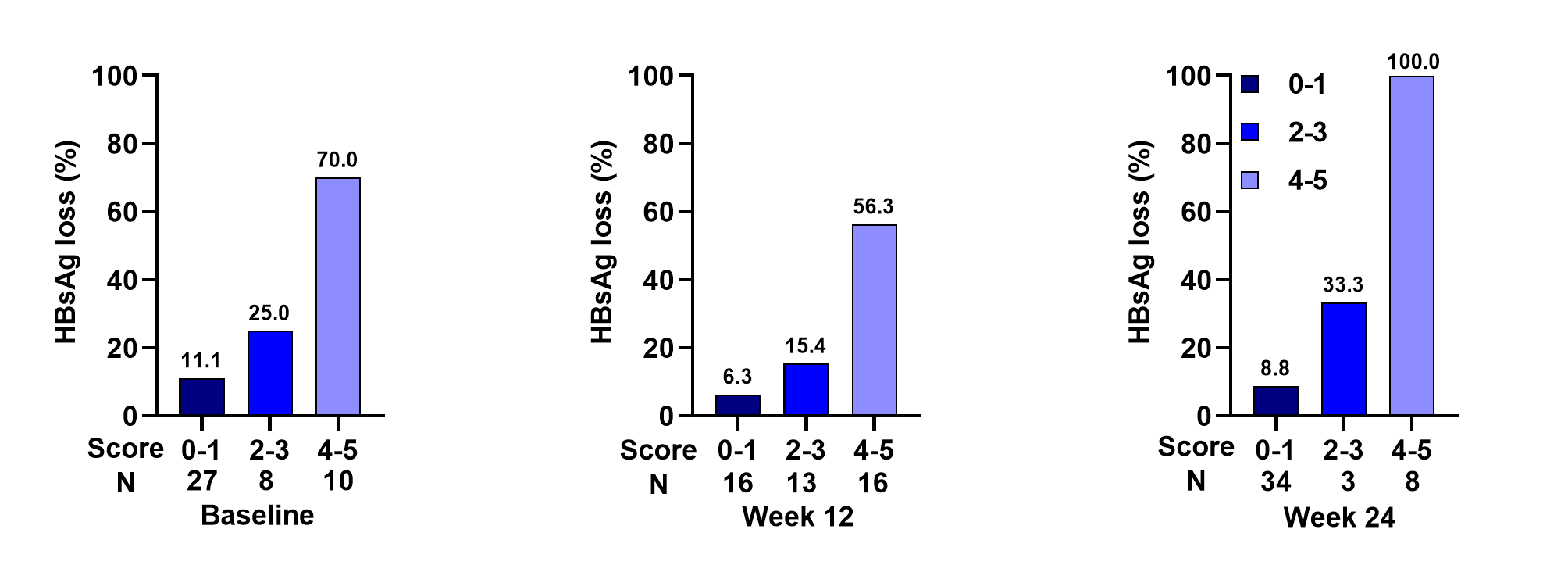


**Figure S4** Application of score model at the initial treatment of patients with PEG-IFNα and NUCs combination.


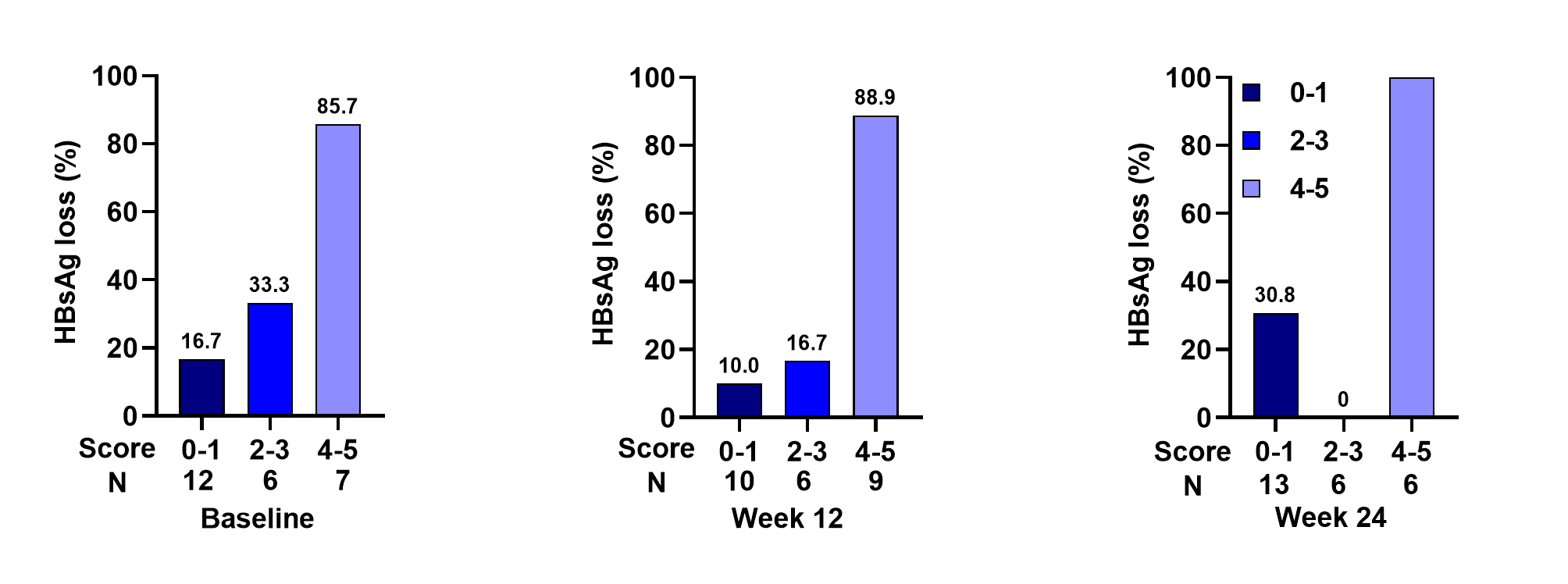


**Figure S5** Application of score model in the experienced treatment of patients with PEG-IFNα monotherapy.


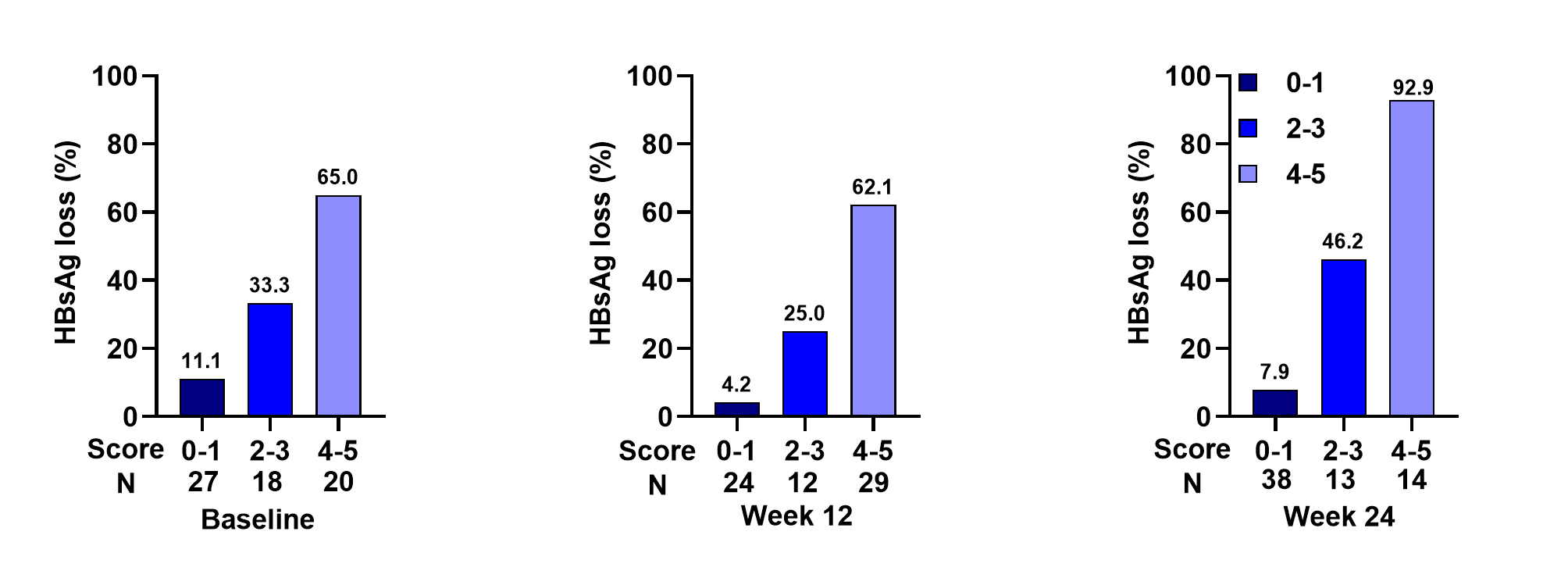


**Figure S6** Application of score model in the experienced treatment of patients with PEG-IFNα and NUCs combination.
